# Synthesis and Anti-*Trypanosoma cruzi* Activity of Diaryldiazepines

**DOI:** 10.3390/molecules20010043

**Published:** 2014-12-23

**Authors:** Júlio César L. Menezes, Luana Beatriz A. Vaz, Paula Melo de Abreu Vieira, Kátia da Silva Fonseca, Cláudia Martins Carneiro, Jason G. Taylor

**Affiliations:** 1 Departamento de Química, ICEB, Universidade Federal de Ouro Preto, Campus Morro do Cruzeiro, CEP, Ouro Preto, MG 35400-000, Brazil; E-Mail: jc_menezes2@hotmail.com; 2 Laboratório de Imunopatologia, Núcleo de Pesquisas em Ciências Biológicas, ICEB II, Morro do Cruzeiro, Universidade Federal de Ouro Preto, Ouro Preto 35400-000, Brazil; E-Mails: luanavazz@yahoo.com.br (L.B.A.V.); paulinhamav@gmail.com (P.M.A.V.); katia.fonseca@gmail.com (K.S.F.); claudiamartinscarneiro@gmail.com (C.M.C.)

**Keywords:** diaryldiazepines, *Trypanosoma cruzi*, Chagas disease, epimastigote

## Abstract

Chagas disease is a so-called “neglected disease” and endemic to Latin America. Nifurtimox and benznidazole are drugs that have considerable efficacy in the treatment of the acute phase of the disease but cause many significant side effects. Furthermore, in the Chronic Phase its efficiency is reduced and their therapeutic effectiveness is dependent on the type of *T. cruzi* strain. For this reason, the present work aims to drive basic research towards the discovery of new chemical entities to treat Chagas disease. Differently substituted 5,7-diaryl-2,3-dihydro-1,4-diazepines were synthesized by cyclocondensation of substituted flavones with ethylenediamine and tested as anti-*Trypanosoma cruzi* candidates. Epimastigotes of the Y strain from *T. cruzi* were used in this study and the number of parasites was determined in a Neubauer chamber. The most potent diaryldiazepine that reduced epimastigote proliferation exhibited an IC_50_ value of 0.25 μM, which is significantly more active than benznidazole.

## 1. Introduction

**Figure 1:**
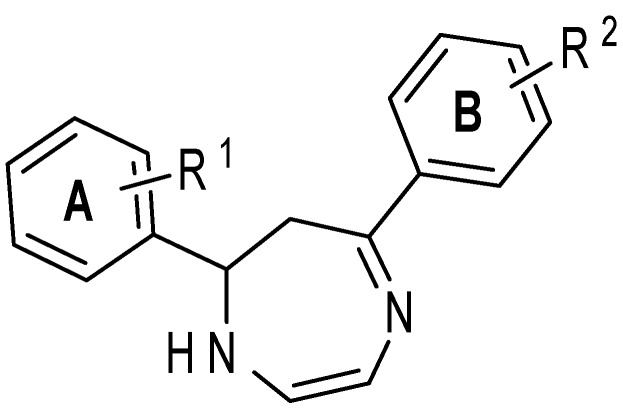
Chemical structure of diaryldiazepines

Seven-membered heterocycles with two nitrogen’s in a 1,4-relationship are classified as diazepines. Diazepines are well known for their unique pharmacological activity and in particular, the 1,4-benzodiazepines are especially recognized for their effects on the central nervous system [1]. In the last 10 years, a variety of 1,4-diazepines have been reported for various biological activities, such as anti-schistosomal activity [2], apoptosis inhibitors [3], Nox4/Nox1 inhibitors [4] and anticonvulsant activity, [5] amongst others. In the specific case of diaryldiazepines, antiproliferative and cytotoxic activities were demonstrated *in vitro* against several human leukemic cells, such as Jurkat, HL60, MOLT3, NCEB-1 and K562 [6]. However, in general, diaryldiazepines have been underexplored for other types of diseases and this is surprising given the possibility to modulate its activity by incorporating different substituents on the phenyl rings (Figure 1).

**Figure 2:**
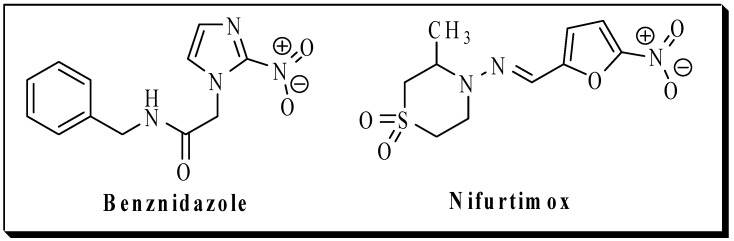
Chemical structures of benznidazole and nifurtimox.

The present work is part of our ongoing medicinal chemistry project funded by the Brazilian government, which aims to drive basic research towards solving neglected tropical diseases (NTD). American trypanosomiasis, also known as Chagas disease [7], is included in the NTD list and is endemic to 21 Latin American countries [8,9]. The protozoan hemoflagellate *Trypanosoma cruzi* is the etiologic agent responsible for Chagas disease. The disease is a so-called “neglected disease” because health markets in affected countries are insufficient to attract pharmaceutical industry to invest in research and development. Thus, the biggest obstacle to treating the disease has been the discovery and development of new chemical entities able to meet this deficit in innovation. The two drugs currently available for treating Chagas disease are nifurtimox and benznidazole, which are chemically related nitro-heterocycles (Figure 2). 

Unfortunately, they both result in many significant side effects such as oedema, fever, rash, agranulocytosis and neurotoxicity. In comparison with benznidazole, not only are the side effects of nifurtimox more severe, it has lower efficacy and so the commercialization of nifurtimox was suspended in Brazil, Argentina, Chile and Uruguay in the 1980s [10]. Additionally, the therapeutic effectiveness of nifurtimox and benznidazole is dependent on the type of *T. cruzi* strain, since certain strains are more resistant than others [11]. Thus, due to the limitations of these treatments, they are not ideal drugs and this drives the search for new more efficient compounds. 

**Figure 3:**
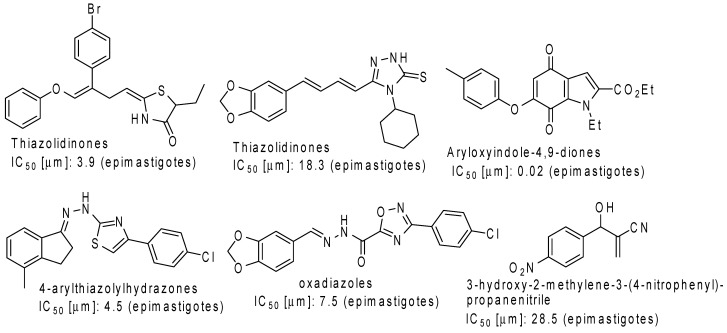
Compounds with anti-*T. cruzi* activity.

A number of studies focused on the synthesis and *in vitro* activity of biologically active compounds against *T. cruzi* have been reported in the literature. Recent examples include thiazolidinones [12], 1,2,4-triazole-3-thiones [13], aryloxyindole-4,9-diones [14], 4-arylthiazolylhydrazones [15], oxadiazoles [16] and Morita–Baylis–Hillman adducts [17]. Promising results are indicated by potencies that are either equal to or better than benznidazole with varying degrees of mammalian cell toxicity observed depending on the compound class (Figure 3). The aryloxyindoles stand out for their high potency but in general, IC_50_ values in the range of 4–30 μM have been considered promising candidate leads.

In order to meet this deficit in new drug candidates for Chagas disease, we have prepared and tested against *T. cruzi* six diaryldiazepines analogous.

## 2. Results and Discussion

**Scheme 1:**
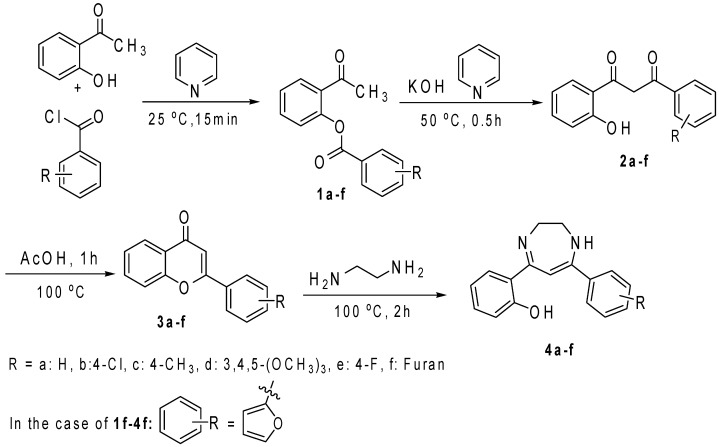
Synthesis of Diazepines **4a**–**f**.

The synthesis of the target diazepine was commenced with the esterification of 2-hydroxyacetaphenone with substituted benzoyl chlorides as illustrated in Scheme 1. 

A Baker-Venkataraman rearrangement induced by KOH affords 1,3-diketones **2a**–**f**, which undergo condensation under refluxing acetic acid to provide flavones **3a**–**f**. All ester, 1,3-diketone and flavone intermediates were confirmed by comparison of their melting point and NMR spectral data with literature values. All data for these intermediates were in complete accordance with literature values. Flavones **3a**–**f** reacted with ethylenediamine to afford the required diaryldiazepines in reasonable yields and high purity. Their spectroscopic data were in accordance with those previously reported [6]. Only diazepine **4d** is novel and was therefore fully characterized by FT-IR, NMR and Mass Spectrometry. To evaluate the possible influence of substituent effects, we prepared diaryldiazepines which either contained an electron donating or withdrawing group. 

Once the final products were purified and fully characterized, we carried out *in vitro* bioassays using epimastigotes forms (*i.e*., the form present in the midgut vector) of Y-strain *T. cruzi*, which provided the inhibitory concentration as summarized in Table 1.

**Table 1:** Trypanocidal activity of diazepines **4a**–**f** against the epimastigotes forms of Y-strain *T. cruzi*.

Compound	Trypanocidal Activity IC_50_ (μM)
Benznidazole	10.8
**4a**	10.6
**4b**	0.25
**4c**	34.2
**4d**	26.8
**4e**	4.2
**4f**	Not Active

IC_50_: Represents the concentration required to give 50% inhibition.

In Brazil, benznidazole is the only therapy used in etiological treatment with direct action against intracellular trypomastigotes of the *Trypanosoma cruzi* parasite. Precisely for this reason, it is the only drug used as a standard control in our experiments. However, due to numerous side effects caused by this drug and resistance build up by different strains of the parasite, several compounds have been studied in the search for new treatments [12,13,14,15,16,17]. Herein, 6 Diazepines compounds were assayed directly against the epimastigotes forms of *T. cruzi*.

Among the diazepines tested, **4a**, **4b** and **4e** were found to be the most potent trypanosomicides against epimastigotes and in particular, diazepine **4b** was 40 times more potent than our positive control, benznidazole. The furan bearing diazepine was essentially inactive and it was therefore not possible to calculate an IC_50_ for **4f** with any statistical confidence due its low activity. No cytotoxicity was observed for this same compound against human leukemic cancer cell lines [6]. 

Diazepine **4b** showed 78% inhibition at the lowest concentration tested and this result highlights the remarkable effect that the chloro group has on the activity of the 5,7-diaryldiazepine. In contrast, the presence of electron donating methoxy groups are slightly unfavourable towards anti-trypanocidal activity. Our results contrast to those of Ramajayam and collaborators [6] in which cytotoxicity activity of 5,7-diaryldiazepines was highest for the methyl group bearing diaryldiazepine. Similarly, electron-donating groups *i.e.*, the methoxy substituents, lowered cytotoxicity. In the present study, these analogues were also less active against *T.cruzi.*

Ramajayam and collaborators found that antiproliferative activity for halogen group bearing diaryldiazepines was moderate, but from the data obtained in the present study, this analogue was significantly more active against *T. cruzi* with compound **4e** being the next most active against epimastigotes of *T. cruzi*. In contrast, the IC_50_ for cytotoxicity of **4b** against human leukemic cancer cell lines Jurkat (81 μM), HL60 (>100 μM), Molt3 (64 μM), NCEB-1 (>100 μM) and K562 (65 μM) suggest only moderate antiproliferative activity. Interestingly, the same compound demonstrated higher activity against epimastigotes of *T. cruzi* than the aforementioned compound classes (see introduction) that have been studied in recent years. These initial promising results will direct us in investigating how substituents on both rings of the molecule contribute to the overall activity against the parasite. 

In conclusion, diaryldiazepine **4b** is the most active against epimastigotes of *T. cruzi* and is 40 times more potent than the current medicine, benznidazole that is used to treat Chagas disease in Brazil. Studies are ongoing to investigate the possible biological mechanism of action. 

## 3. Experimental Section 

All commercial reagents were used as received. TLC analyses were performed using silica gel plates, using ultraviolet light (254 nm) or vanillin solution for visualization. Melting points are uncorrected. IR spectra were recorded using samples that were prepared between NaCl plates, or pressed in to KBr discs. For NMR data, the chemical shifts are reported in δ (ppm) referenced to residual protons and ^13^C signals in deuterated chloroform or deuterated DMSO. The coupling constants (*J*) are expressed in Hertz (Hz). Mass spectra were recorded on a Shimadzu LCMS-IT-TOF mass spectrometer. Flavones **3a**–**f** were prepared according to literature methods [18].

### 3.1. Chemistry

General method for the preparation of 5,7-diaryl-2,3- dihydro-1,4-diazepine derivatives is based on the protocol described by Ramajayam and co-workers [6] and involves refluxing a mixture of flavone (5–15 mmol) in ethylenediamine (20 mL) for 2 h. The cooled mixture was poured into ice water and the yellow precipitate was filtered and finally recrystallized from methanol to give analytically pure samples.

*5-(2-Hydroxyphenyl)-7-phenyl-2,3-dihydro-1H-1,4-diazepine.* Compound **4a** was obtained as a yellow solid in 57% yield. MP. 209–211 °C (Lit MP. 208–210 °C [6]); (300 MHz, DMSO-d_6_) δ: 8.0 (1H, brs), 7.38–7.58 (7H, m), 7.0 (1H, t, *J* 8.5), 6.74 (1H, d, *J* 8.0), 6.45 (1H, t, *J* 8.0), 5.6 (1H, s), 3.8 (2H, bs), 3.6 (2H, bs); δ_C_ (75 MHz, DMSO-d_6_): 170.2, 167.2, 158.9, 140.4, 136.2, 132.5, 129.6, 127.19, 127.8, 121.2, 117.1, 114.1, 86.1, 50.4, 49.3. 

*5-(2-Hydroxyphenyl)-7-(4-chlorophenyl)-2,3-dihydro-1H-1,4-diazepine.* Compound **4b** was obtained as a yellow solid in almost quantitative yield. MP. 238–240 °C (Lit MP. 243–245 °C [6]); υ_max_ (cm^−1^): 1570, 1529, 1490, 1439, 1318, 1289, 1260, 1143, 837, 751; δ_H_ (300 MHz, DMSO-d_6_): 7.66–7.45 (5H, m), 7.08 (1H, t, *J* 7.8), 6.56 (1H, d, *J* 8.2), 6.38 (1H, t, *J* 7.0), 5.61 (1H, s), 3.65 and 3.95 (4H, bs); δ_C_ (75 MHz, DMSO-d_6_): 170.8, 168.2, 157.8, 137.7, 135.2, 132.7, 129.8, 129.3, 129.0, 128.7, 128.0, 121.7, 116.9, 113.2, 86.6, 49.9, 49.0, 31.1.

*5-(2-Hydroxyphenyl)-7-(4-methylphenyl)-2,3-dihydro-1H-1,4-diazepine.* Compound **4c** was obtained as a yellow solid in 47% yield. MP. 233–235 °C (Lit.MP. 235–237 °C [6]); υ_max_ (cm^−1^): 3221, 2922, 1604: δ_H_ (300 MHz, DMSO-d_6_): 8.14 (1H, s), 7.59–7.51 (3H, m), 7.28 (2H, d, *J* 8.0), 7.07 (1H, t, *J* 7.0), 6.54 (1H, d, *J* 8.5), 6.37 (1H, t, *J* 8.0), 5.62 (1H, s), 3.81 (2H, s), 3.56 (2H, s), 2.35 (3H, s); δ_C_ (75 MHz, DMSO-d_6_): 170.8, 168.2, 158.9, 140.4, 136.2, 132.5, 129.6, 127.9, 127.8, 121.6, 117.1, 113.1, 86.1, 50.0, 49.0, 21.3.

*5-(2-Hydroxyphenyl)-7-(3,4,5-trimethoxyphenyl)-2,3-dihydro-1H-1,4-diazepine.* Compound **4d** was obtained as a yellow solid in 43% yield and is a novel compound. MP. 229–230 °C; υ_max_ (cm^−1^): 1582, 1531, 1499, 1309, 1244, 1124, 1009, 756; δ_H_ (300 MHz, DMSO-d_6_): 8.12 (1H, brs), 7.64 (1H, d, *J* 8.5), 7.08 (1H, t, *J* 7.8), 6.56 (1H, d, *J* 8.3), 6.42 (1H, t, *J* 7.0), 5.67 (1H, s), 3.84 (6H, s), 3.69 (3H, s), 3.54 (4H, bs); δ_C_ (75 MHz, DMSO-d_6_): 172.5, 170.5, 158.7, 153.1, 134.5, 132.4, 128.2, 121.4, 117.3, 113.2, 105.5, 86.2, 60.5, 56.5, 50.2, 49.1; MS *m*/*z* (EI): calcd. for C_20_H_22_N_2_O_4_ 354.1580, found 354.1571. 

*5-(2-Hydroxyphenyl)-7-(4-fluorophenyl)-2,3-dihydro-1H-1,4-diazepine.* Compound **4e** was obtained as a yellow solid in 49%. MP. 235–236 °C (MP. 234–236 °C Lit. [6]); υ_max_ (cm^−1^): 1603, 1531, 1499, 1440, 1318, 1259, 1224, 1168, 1144, 845, 783, 751; δ_H_ (300 MHz, DMSO-d_6_): 7.68 (2H, t, *J* 8.7), 7.59 (1H, d, *J* 8.4), 7.3 (2H, t, *J* 8.7), 7.08 (1H, t, *J* 6.9), 6.55 (1H, d, *J* 9.3), 6.38 (1H, t, *J* 8.1), 3.65 and 3.95 (4H, bs); δ_C_ (75 MHz, DMSO-d_6_): 171.3, 168.4, 165.5 and 162.2 (d, *J*_CF_ 247.5), 158.4, 135.639 and 135.601 (d, *J*_CF_ 2.9), 132.9, 130.6, 130.5, 130.5, 128.3, 122.0, 117.1, 116.3 and 116.0 (d, *J*_CF_ 59.7), 113.3, 86.6, 50.0, 49.2. 

*5-(2-Hydroxyphenyl)-7-(2-furyl)-2,3-dihydro-1H-1,4-diazepine.* Compound **4f** was obtained as a yellow solid in 77% yield. M.P. 175–178 °C (Lit MP. 168–170 °C [6]); υ_max_ (cm^−1^): 1602, 1537, 1511, 1329, 1301, 1253, 1149, 1023. δ_H_ (300 MHz, DMSO-d_6_): 7.87 (1H, s), 7.66 (1H, d, *J* 8.4), 7.21 (1H, d, *J* 3.3), 7.1 (1H, t, *J* 8.1), 6.68–6.66 (1H, m), 6.56 (1H, d, *J* 9.3), 6.44 (1H, t, *J* 8.1), 6.03 (1H, s), 3.65 and 3.95 (4H, bs); δ_C_ (75 MHz, DMSO-d_6_): 171.1, 168.5, 150.1, 147.1, 145.7, 132.9, 128.1, 121.9, 117.2, 113.5, 113.2, 111.7, 83.0, 50.0, 48.8. 

### 3.2. Pharmacology

The IC_50_ values of the drug concentration (μM) necessary to kill 50% of the parasites, was obtained by linear regression analysis.

### 3.3. Anti-Trypanosoma cruzi Activity

In this study, epimastigotes of the Y strain from *T. cruzi* were used. Epimastigotes obtained in exponential growth phase were washed with LIT medium 2 times in sterile PBS at pH 7.2 (1500 g) for 10 min at 4 °C. The number of parasites was determined in a Neubauer chamber. Next, the parasites were suspended in LIT medium, which was further supplemented with 10% FBS (Fetal Bovine Serum inactivated at 56 °C), and the concentration of epimastigotes adjusted to 5 × 10^6^ epimastigotes/mL.

With the goal of screening samples with possible biological activity against *T. cruzi*, compounds were incubated in 48 well Nunc plates containing 800 μL of the suspension of parasites and 200 μL of the test compounds at different concentrations (40, 20, 10 and 1.6 μg/mL) diluted in sterile DMSO for 72 h in duplicate. As a negative control, the parasites were incubated in the absence of test compound and in the presence of 1% DMSO. Benznidazole (10 μg/mL) was used as positive control in tests against *T. cruzi*. The activity was determined by counting in a Neubauer chamber and subsequent statistical evaluation. The results are expressed as IC_50_. The tests were repeated 2 times with the objective of evaluating the maintenance of the activity of the compounds and reproducibility of results.
